# A Contribution of Beef to Human Health: A Review of the Role of the Animal Production Systems

**DOI:** 10.1155/2016/8681491

**Published:** 2016-02-16

**Authors:** Dario Pighin, Adriana Pazos, Verónica Chamorro, Fernanda Paschetta, Sebastián Cunzolo, Fernanda Godoy, Valeria Messina, Anibal Pordomingo, Gabriela Grigioni

**Affiliations:** ^1^Food Technology Institute-INTA, Morón, B1708WAB Buenos Aires, Argentina; ^2^The National Council for Scientific and Technical Research (CONICET), Rivadavia 1917, C1033AAJ Buenos Aires, Argentina; ^3^Morón University, Cabildo 134, Morón, B1708JPD Buenos Aires, Argentina; ^4^CINSO-CITEDEF, UNIDEF (Strategic I & D for Defense), CONICET-Ministry of Defense, Juan Bautista de la Salle 4970, Villa Martelli, B1603ALO Buenos Aires, Argentina; ^5^Experimental Station INTA Anguil, National Route, No. 5, Km. 580, 6326 La Pampa, Argentina

## Abstract

Meat and meat products constitute important source of protein, fat, and several functional compounds. Although beef consumption may implicate possible negative impacts on human health, its consumption can also contribute to human health. Quality traits of beef, as well as its nutritional properties, depend on animal genetics, feeding, livestock practices, and* post mortem* procedures. Available data show that emerging beef production systems are able to improve both, quality and nutritional traits of beef in a sustainable way. In this context, Argentina's actions are aimed at maximising beef beneficial effects and minimising its negative impact on human health, in a way of contributing to global food security.

## 1. Introduction

Meat is an important part of human diet with strong implications in health, economy, and culture worldwide. Meat production involves numerous domestic species, depending on many factors like religious and cultural beliefs, convenience, availability, and so forth [1].

It is well established that meat has several key nutritional factors, like lipids, proteins with high biological value, trace elements, and vitamins [2, 3]. Meat quality intrinsic characteristics such as colour, flavour, tenderness, texture, juiciness, and odour as well as its nutritional properties depend on animal genetics, feeding, and livestock practices and on the* post mortem* processes that take place during the conversion of muscle into meat [4].

Due to the stated reasons, beef consumption as part of balanced diets in developing regions will promote nutrition security. Thomas et al. [5] stated the importance of animal agriculture not only for the production of high quality proteins but also for sustaining rural livelihoods and possibly contributing to food security. Nevertheless, it is important to remark that since energy and protein transformation efficiency in ruminants is very low, food security can be effectively promoted only if feeds given to the animals are not in competition with humans.

The World Food Summit of 1996 defined food security as existing “when all people at all times have access to sufficient, safe, nutritious food to maintain a healthy and active life.” Commonly, the concept of food security is defined as including both physical and economic access to food that meets people's dietary needs as well as their food preferences. Food security is a complex sustainable development issue, linked to health through malnutrition, but also to sustainable economic development, environment, and trade.

Food and Agriculture Organization (FAO) pointed out that the quality of diets has also been improved. In developing regions, several improvements were observed over the last two decades. For example,* per capita* availability of fruits and vegetables, livestock products, and vegetable oils increased by 90, 70, and 32 percent since 1990–92, respectively. A 20% increase in protein availability per person was also noted. FAO stated that these enhancements were not fully seen in Africa or Southern Asia. In these regions, diets remain imbalanced and heavily dependent on cereals and roots and tubers. These monotonous diets often comprised negligible quantities of meat, fish, or ascorbic acid. As a consequence, they typically contained a preponderance of foods that inhibit ferric absorption. It should be emphasized that absorption of micronutrients is strongly influenced by the combination of foods eaten in a given meal [6]. Moreover, increasing fat content of diets often facilitates absorption of provitamin A, carotenoids, and vitamin A.

Meat consumption may also represent some risks to human health. Depending on several factors, many reports warn against its metabolic deleterious effects specially linked to cholesterol saturated fatty acids (SFA) levels. Low polyunsaturated fatty acids (PUFA) levels, or inappropriate SFA/PUFA or PUFA n-6/PUFA n-3, had been represented as an inconvenient in usual meat consumption.

Also, fresh meat is a highly perishable product due to its biological composition. Several factors such as storage temperature, packing conditions, endogenous enzymes, moisture, light, and microorganisms can affect shelf life and freshness. In this sense, meat processing and preservations technologies play essential roles in food security, in order to supply the expanding populations with sufficient quantities of good-quality and affordable meat products.

Several authors have reported methods and technologies to be applied in fresh meat with the aim of extending meat shelf life [7]. One of the common processes used in meat preservation is concerned with inhibiting microbial spoilage, and applying these methods deteriorative changes such as colour and oxidative process should be minimized [8]. Zhou and coauthors [7] presented an extended review comprising current methods and technologies for fresh meat preservation, their applications, and implications for extending meat shelf life.

This review attempts to summarize the recent progress in scientific research regarding the effect of agricultural practices, with special focus on Argentina's actions, on the improvement of nutritional value and quality characteristics of beef as a contribution to improve beef healthiness and global food security. The paper is organized in three sections that provide an outline of the lipids and proteins in beef and an overview of beef production systems in Argentina as a particular case for maximising its beneficial effects and minimising its negative impacts.

## 2. Lipids

### 2.1. Importance of Lipids in the Diet

In the last decades, there has been an increased interest in ways to manipulate the fatty acid composition of meat, since it is seen to be a major source of fat in the human diet. Human health recommendations include a fat intake of 15–30% of total energy intake [9]. Since the relative amounts of polyunsaturated fatty acids (PUFA) and saturated fatty acids (SFA) seem to play a key role in a healthy and balanced diet, a fatty acid intake up to 10% of saturated fatty acids (SFA) and a ratio of PUFA to SFA (P : S ratio) above 0.4 are recommended. Among PUFA, the ratio n-6 : n-3 should be under 4 [10].

Since SFA, specially 12:0, 14:0, and 16:0, have been traditionally associated with increased level of cholesterol in blood stream and, consequently, with coronary heart disease (CHD) and cardiovascular disease (CVD), their deleterious metabolic effects are questioned at present. A recent meta-analysis of epidemiologic studies carried out by Siri-Tarino et al. [11] found no significant evidence for concluding that SFA are associated with increased risk of coronary heart disease or cardiovascular disease. Nevertheless, there is still an important emphasis in reducing SFA since the beneficial effects associated with the substitution of SFA with n-3 PUFA [12].

More recently, conjugated linoleic acid (CLA) and long chain PUFA (n-3) contents, eicosapentaenoic acid (EPA) and docosahexaenoic acid (DHA), have also become imperative due to their multiple healthy metabolic effects, like reduction of the risk of cardiovascular disease, proper brain and visual development in fetal life, and maintenance of neural and visual tissues throughout life [13–17]. Recent studies have stated a beneficial effect of the n-3 fatty acid *α*-linolenic acid, ALA, at the low dose of 4.4 g per day, a perfectly achievable dose by means of regular consumption of ALA-rich sources [18].

Beef and other ruminant products constitute important dietary source of CLA, especially cis-9, trans-11 isomer, identified as an important health promoter factor including antitumoral and anticarcinogenic activities [19]. Biological effects have been widely studied also for the trans-10, cis-12 isomer, identified as an important antiobesity factor [20]. Beef also contains* trans-*fatty acids (TFA), being vaccenic acid,* trans*-11 18:1, its most representative one. An intake of TFA lower than 1% of dietary energy has been recommended [21]. Nevertheless, in the last years, TFA became also very important since its potential protective properties against the development of coronary heart diseases [19]. Thus, at present, a great deal of effort is being done in differentiating natural from industrial TFA.

### 2.2. Factors That Modify Beef Lipid Content and FA Profile

Fat content and FA composition of beef may differ according to breed or genotype, the feeding background, and the muscle considered. Although beef usually has a P : S ratio around 0.1, its ratio n-6 : n-3 PUFA is particularly beneficial (around 2), especially from animals fed with grass containing high levels of PUFA n-3 [22]. Both, genetic and nutritional approaches have been widely studied in relation to FA profile of beef. In this regard, it is recognized that genetic factors provide smaller differences than nutritional ones [23]. Genetic factors reflect differences in gene expressions of enzymes involved in fatty acid synthesis. Thus, a particular relationship between fatness and FA profile has been stated [13]. As the content of SFA and MUFA increases with increasing fatness, the relative proportion of PUFA and the consequent P : S ratio decrease with it. Hence, lean meat with low fat content, less than 1%, would contain a healthier P : S ratio than high fat meat [24].

Regarding the effect of the diet, that is, the production system, it has been demonstrated that ruminants meat contains beneficial ratio of n-6 : n-3 PUFA, that is, below 4, especially when they had consumed grass-based diets [22]. Beef from pasture-finished steers has greater levels of n-3 PUFA when compared to concentrate-finished steers [25, 26]. Similar results were also found in pasture-finished bulls [27]. Moreover, fresh and conserved (silage and hay) grass presents different effects on the n-3 PUFA deposition into the muscle. Thus, higher levels of n-3 PUFA in the muscle of cattle fed fresh grass has been demonstrated than cattle fed hay [13]. An overall and compact view about the effect of different diets, from different production systems, is shown in Table 1. In this table, data regarding lipid content of* LD* muscle and its FA composition is compiled as a general example of the major effects of animal feeding on beef quality.

It is important to remark that muscle lipids are distributed in different compartments or fractions. Thus, neutral fraction, usually characterized by high proportions of SFA and monounsaturated fatty acids (MUFA), is located along the muscle fibres, in the interfascicular area, and in cytosolic droplets into the muscle cells. This FA fraction is easily influenced by diet composition despite the saturation occurring at ruminal level. On the other hand, the polar fraction, composed by phospholipids and usually characterized by high proportion of PUFA, is located in the cell membranes. Due to the higher proportion of phospholipids, genetically lean breeds show higher levels of PUFA [34]. This FA fraction can be less influenced by diet and its content is independent of the total fat content [35]. Moreover, the fatty acid composition might also display a muscle effect, since muscle fiber type can affect fatty acid composition: red, oxidative, muscles have higher proportion of phospholipids and, therefore, contain higher levels of PUFA than white, glycolytic, muscles [22]. In this regard, Enser et al. [36] have reported a P : S ratio in* Gluteo biceps* muscle, oxidative, red muscle, significantly higher than in the whiter* Longissimus* muscle in grass-fed steers.

### 2.3. Influence of FA Profile on Beef Quality Traits

Fatty acids are also involved in several physicochemical properties of meat, contributing not only to the nutritional attributes of beef but also to the physicochemical ones. Thus, different fatty acids (saturated or unsaturated) show different melting points, which in turn affects the firmness or softness of meat fat. On the other hand, the presence and degree of double bonds in the fatty acid structure affect the oxidation susceptibility, which in turn regulates the shelf life of meat [22].

Marbling fat, total fatty acid content in muscle, has been long recognized as a quality factor of meat. It has been also positively associated with juiciness and tenderness, although its contribution is indirect. It has been proposed that neutral lipids in fat cells could have a physical effect in separating muscle fiber bundles. It has been proposed that lipids could also retain water in the muscle structure leading to increased water holding capacity and associated juiciness [22].

Flavour development during cooking also depends on the PUFA content of meat fat which leads the volatile compounds generation. Nevertheless, the desirable increase of PUFA in beef has the disadvantage of increasing the susceptibility to oxidation. In this regard, it has been stated that lipid oxidation is the major cause of colour, flavour, and nutritional value deterioration in meat [37]. Consequently, much effort has been made to protect these unsaturated structures by means of antioxidants elements like vitamin E [38–40]. Regarding this issue, it has been demonstrated that pasture production systems not only increase n-3 PUFA in beef but also increase vitamin E, *α*-tocopherol, carotenoids, and flavonoids, extending its lipid stability and colour shelf life [39, 41]. Grain production systems may improve the beef colour stability and shelf life by supplementing the animal diet with natural antioxidants, that is, *α*-tocopherol [42].

Cooking procedures may affect the fat content and FA profile of both pasture- and feedlot-finished beef in a similar way [30]. Interesting data regarding the effect of cooking methods upon the nutritional quality of beef intramuscular fat has also been recently published. The effect of boiling, microwaving, and grilling on the composition and nutritional quality of beef intramuscular fat has been investigated. Results obtained demonstrated that the content of total lipids increased, by means of a concentration effect, with the cooking time and internal temperature reached [30]. The major changes in FA composition resulted in higher percentages of SFA and MUFA and lower levels of PUFA in cooked meat. CLA had revealed great stability to thermal processes [30].

## 3. Proteins

### 3.1. Importance of Proteins in the Diet

Meat muscle composition is approximately 19% proteins, being 11.5% structural proteins (myofibrillar), 5.5% soluble sarcoplasmic proteins, and 2% connective tissue (collagen and elastin), and 2.5% fat, dispersed among protein fibers [43]. The protein content is modified in cooked meat due to water loss through the cooking process. These proteins become highly digestible (94%) [44].

Paddon-Jones and Leidy [45] stated that red meat is a source of high quality protein and highly bioavailable iron to enhance vitality. Several authors have reported the ability of high quality proteins to promote weight loss, prevent weight gain and weight regain in adults [46–48], reduce fat mass [49], and protect against reductions in lean body mass [50–53]. Losses in high quality protein, especially in older adults, cause sarcopenia (degenerative loss of skeletal muscle mass) and sarcopenic obesity by replacing lost skeletal muscle into fat [45, 54, 55]. Consequently, increasing consumption of high quality protein from middle age has been recommended in order to maintain the quality of life associated with adequate muscle mass. Protein content would maintain or increase fat-free mass by favouring a stimulatory effect on muscle protein anabolism in humans [45, 54–56].

Weight loss diets contain higher amounts of protein, which have been shown to be more effective compared to standard protein diets. Moreover, several authors showed a greater overall satisfaction in terms of food palatability, pleasure, and enjoyment in subjects consuming high protein diets as compared to lower protein diets [57–60].

### 3.2. Role of Aminoacids in Human Health

Aminoacids and bioactive compounds are very important molecules to prevent muscle-wasting diseases, that is, sarcopenia, to reduce calorie intake (metabolic syndrome prevention), to control blood pressure homeostasis, via ACE-inhibitory components from the connective tissue, and to maintain the functionality of intestinal environment, through nucleotides and nucleosides of meat [61].

Aminoacids like leucine, isoleucine, and valine are essential for protein synthesis. Leucine supplementation has been shown to increase muscle protein synthesis in older adults [62]. Furthermore, protein ingestion strongly increases muscle protein synthesis rates, effect mainly attributed to the stimulatory effect of essential aminoacids [63]. Beef also contains high amounts of glutamic acid/glutamine (16.5%), arginine, alanine, and aspartic acid.

Phillips [64] reported that senescent muscle is less sensitive to the anabolic properties of aminoacids. Leucine has been reported to stimulate muscle protein synthesis in an insulin dependent and independent manner. Consequently, it has been suggested that increasing the leucine content of meals in the elderly could compensate the decreased muscle protein synthetic response to food intake.

Beef is also rich in branched-chain aminoacids, leading to further metabolic effects. Thus, comparing beef with soya, Phillips [64] has reported greater myofibrillar proteins synthesis, both at rest and after performance of resistance exercise, in those individuals submitted to beef feeding. Moreover, Bhutta [65] stated that meat proteins provide all essential aminoacids (lysine, threonine, methionine, phenylalanine, tryptophan, leucine, isoleucine, and valine) with no limiting aminoacid.

### 3.3. Biopeptides

Bioactive peptides are sequences of 2–30 aminoacids that impart a positive health effect to the consumer when ingested, playing an important role in the prevention of diseases associated with the development of metabolic syndrome and mental health diseases [66].

Meat contains several proteins and peptides with important physiological activities. It has been demonstrated that collagen has a positive influence on the delivery and bioactivity of bone morphogenic protein-2 and ectopic bone formation, enhancing bone healing [67]. Other varieties of beneficial effects on health by meat peptides include antihypertensive, antioxidant, antithrombotic, anticancer, immune modulatory, and antimicrobial activities. In the last years, the possibility of obtaining bioactive peptides from meat proteins by means of different procedures like hydrolysis, cooking, and fermentation has been explored [68].

Some peptides are inactive in the sequence of the parent protein but may have a positive effect once released. A variety of bioactive peptides are naturally occurring in animals or are generated* post mortem* by endogenous enzymes in meat and [69].

At present, there is insufficient information about the physiological functions of beef peptides in both animal and human models. Thus, the study of the beef proteins as precursors of functional biopeptides, in order to develop functional foods and nutraceuticals, remains a great issue to be explored [70].

Although several bioactive compounds in meat, carnosine, anserine, and L-carnitine, have been recently studied, the effect of beef production systems on these bioactive compounds remains almost unexplored. Recent studies [71] demonstrated that preslaughter management can affect the beef content of anserine and carnosine, both in extensive (pasture-based) and in intensive (grain-based) systems. Carnosine beef content also displayed a production system-associated behaviour, with higher levels in pastured-based beef when compared to intensive-based beef [71]. However, Arihara [72] reported that there are still some obstacles in the development and marketing of new functional meat products as these products are unconventional.

## 4. The Argentinean Perspective of Beef Production Systems and Implications on Meat Quality

### 4.1. Argentinean Livestock Production Systems

Argentina is a well-known producer of pasture-fed beef. Traditionally, beef production was based on low-input systems, which combined grazing complemented with grain as energy supplements to provide pasture-finished beef. However, during the last two decades, Argentinean beef production has evolved into a diversification and intensification process of grazing systems as a result of cash crop expansion caused by the increase in grains prices.

In Argentina, the process of producing beef can be explained as divided into two main activities: (a) cow-calf on marginal lands and (b) steers growing and fattening on better soils [73]. At present, one-third of the cow-calf farmers retain calves and rear them on the same farm on grain supplemented pastures or in confinement until slaughter. The remaining two-thirds still produce calves on extensive cow-calf systems [74].

In the last years, less than 2% of rearing and fattening cattle farms of tempered regions practice pure grazing systems. Most farmers combine grain cropping with livestock in mixed systems. A field is normally kept with a legume-based perennial pasture for a 4–6-year period, followed by a period of annual forages and grain cropping. Rotation schemes depend on several factors, like soil quality, technology availability, and economics competence.

More than 70% of beef produced in Argentina is still produced in pasture-based systems, most widely spread in the temperate areas [74]. Those systems are the least energy intensive and rely on adjusted forage chains depending on rainfall, temperature, and soils quality. In rotation with grain cropping, forage chains include legume-based pastures (primarily alfalfa) and small-grain winter annuals crops (rye, oats, ryegrass, and triticale). Most cattle fattening farmers make a strategic use of energy supplement when necessary, being cereals grain (corn and sorghum) the most common supplement. More recently, confinement feeding at final stage of fattening has been introduced by some farmers.

Overall average daily gains of pasture-finished steers are in the 600 to 700 g/day range on 100% grazing systems. Slower cycles on pure grazing systems have lower average body weight daily gain and include feeding restriction in winter followed by compensatory growth of cattle in spring and summer. Growth continues at moderate rates during a second winter period, targeting full finishing the following spring or summer.

Confinement feeding was lately introduced as strategy to remove animals from grain cropping lands. Confinement feeding takes place at the end of grazing periods (finishing lots) or previous to the initiation of pasture programs, stocker phase, also called “beginning lots.” Feedlots are more efficient in terms of land occupation but much less in terms of environmental impact, competition with human diet, and meat safety.

**Figure 1:**
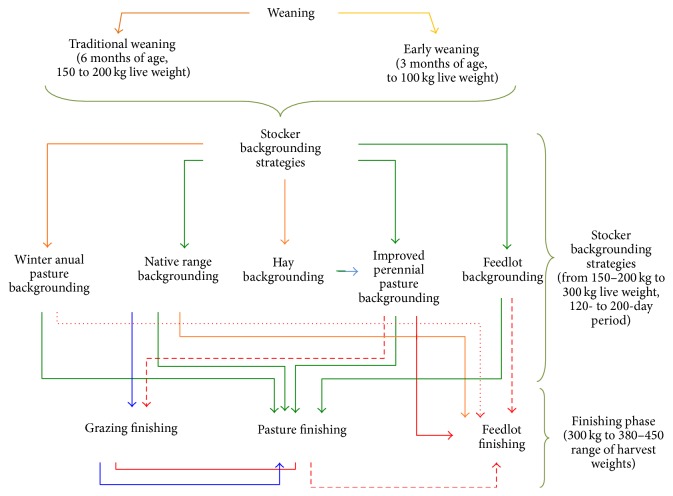
Illustrative platform of main beef production systems in Argentina.

A brief scheme of the Argentinean beef production platform mentioned above is presented in Figure 1 (adapted from [75]).

### 4.2. Argentinean Market of Beef

In Argentina, market preferences for freshness and tenderness led the adjustments for beef quality. Argentinean consumers have a preference for fresh and lean beef. Additionally, the market does not have a taste for aged beef and most packing plants geared to the domestic market are not prepared for stocking beef beyond week. Therefore, beef tenderness has been accomplished by processing young and light, early maturing, easy fattening animals with body condition scores of 3–5.

The increasing world interest in tenderness, flavour, and lipid profiles has pushed research nationwide. Research has largely focused on attributes of beef generated on different feeding and grazing strategies. Previous studies [76–78] had reported that pasture-finished beef is less tender than concentrate-finished. Nevertheless, Argentinean studies [28, 79] have not detected such differences, by means of WB shear force, between grain and grass-fed beef finished to a similar fatness endpoint. Similarly, Realini et al. [80] found no differences between steaks from concentrate- and pasture-finished beef in Uruguay, despite differences found in carcass weight, fatness, and temperature during chilling. Likewise, Duckett et al. [81], French et al. [82], and Mandell et al. [83] found no differences in WB shear force ratings between feedlot-fed and pasture-fed beef in the US, when animals were finished to similar age or fatness.

Bearing in mind that the type of forage could affect beef characteristics, research on pasture finishing on different forages has been carried out. Pordomingo et al. [29, 84] finished steers on winter annuals (triticale, cereal rye, and wheat) or alfalfa to assess beef characteristics. The authors reported no effects of forage source on WB shear force, back-fat thickness, or hot carcass yield. Alfalfa-finished animals had in average more IMF than the grass-finished ones. Wheat-finished animals had a similar content to alfalfa-finished ones. Cereal rye yielded the animals with less desirable profiles compared with the other treatments. The meat quality parameters of shear force, panel tenderness scores, and colour were similar to those reported for feedlot-finished animals in other studies, in both Argentina [28, 85] and Uruguay [80].

Argentinean research suggests that pasture-finished beef is likely to be leaner and lower in cholesterol concentrations than feedlot beef [85–90]. Conversely, Rosso et al. [86] had reported the opposite for IMF content, considering animals of different age in their study. In turn, Volpi Lagreca et al. [28] reported no differences in IMF and back-fat content in feedlot- versus pasture-finished animals when fattened to a similar back-fat thickness and live weight endpoint.

Martínez Ferrer et al. [91] reported a trend (*P* = 0.11) towards a higher proportion of SFA in pasture-based beef, due to an increase in C18:0 (*P* = 0.047), C14:0 (*P* = 0.12), and C16:0 (*P* = 0.37), from steers finished at 10 mm of subcutaneous fat depth. Most studies point out that the proportion of SFA would not be altered by feedlot fattening. Consistently, the highest PUFA concentrations were observed in pasture-based beef [29, 79, 85]. Volpi Lagreca et al. [28] detected greater PUFA n-6 concentrations in feedlot compared with pasture finishing (Table 1).

Most Argentinean studies which have finished steers on a starch-based diet [28, 79, 85, 90–93] reported greater n-6/n-3 ratios compared with pasture diet (Table 1). It has been also demonstrated that the addition of supplemental grain on pasture systems would increase this ratio. On the other hand, studies of Martínez Ferrer et al. [90, 94] and Depetris et al. [95] pointed out that pasture grazing strongly ameliorates the effects of starch feeding on lipids profiles.

Carryover effects of supplementation [96] or feedlot backgrounding [29] on lipids profiles of pasture-finished cattle could be expected. The last authors compared feedlot backgrounding on diets with increasing content of hay with pasture backgrounding on pasture-finished heifers. Results demonstrated that grazing during 132 days after feedlot backgrounding removed only partially the effect of the starch-rich feedlot diets on the fatty acid profile of* Longissimus dorsi* of heifers. Omega 3 fatty acid concentrations remained higher for animals backgrounded on pasture or a 100% hay diet, compared to 40 and 70% hay diets.

Regarding CLA levels, Latimori et al. [79, 85] and Martínez Ferrer et al. [90, 94] have reported increased levels (3-fold) of CLA in pasture-finished beef, when compared to feedlot-finished beef of steers. Thus, while CLA concentration in IMF from animals grown and finished on alfalfa pastures is likely to be in the range of 0.7–0.8%, grain supplementation on pasture would tend to reduce CLA content. Nevertheless, this CLA content of beef would double the level when compared to beef from grain or corn silage-based feedlot diets. Based on this evidence, it could be suggested that CLA content of beef would not be greatly affected by limited energy supplementation of grazing cattle on leguminous pastures.

Regarding the effect of feedlot feeding during a stocker program, Pordomingo et al. [29] noted that pasture-finished heifers, backgrounded in feedlot during 104 days, resulted in CLA beef contents below 0.5%. No differential effects given to energy content of the feedlot-fed diet were detected. Results from this study suggest that systems that pursue CLA enriched beef would need to consider the nature of the diet from the early stages of the growing-finishing program.

## 5. Conclusions

Adequate management of beef production systems would constitute one of the major tools to improve beef quality in a sustainable way. Argentinean production systems may promote food security by means of animal feeding mainly based on feeds not used in human nutrition. They have demonstrated an improvement of beef healthiness, minimizing several negative effects associated with beef consumption, while containing the environmental impact.

Our thought is that research efforts must be stressed to deepen the current knowledge regarding the contribution of animal production systems to maintain beef safety and its biological composition during longer periods of time.

## Figures and Tables

**Table 1:** Lipid content and fatty acid composition reported of beef muscle from steers (British and crossbred) finished in different and contrasting production systems.

	Production system			Reference
	Pasture	Supplementation	Feedlot	
IMF (%)	2.86 b	4.09 a	3.85 a	[25]
	4.96		4.52	[28]
	2.83			[29]
	0.98 b		1.30 a	[30]
	2.80 b		4.40 a	[31]
	2.12 b		3.61 a	[32]

SFA (% total FA)	38.40 a	37.85 a	35.33 b	[25]
	46.61		45.80	[28]
	43.1			[29]
	38.76		39.27	[30]
	48.80 a		45.10 b	[31]
	42.45 b		43.43 a	[32]

MUFA (% total FA)	37.74 b	40.89 a	40.77 a	[25]
	41.63		37.35	[28]
	30.2			[29]
	24.69 b		34.99 a	[30]
	42.50 b		46.20 a	[31]
	43.87 b		47.89 a	[32]

PUFA (% total FA)	7.95 a, b	7.50 b	9.31 a	[25]
	5.58 b		10.12 a	[28]
	8.73			[29]
	28.99 a		19.06 b	[30]
	3.41 a		2.77 b	[31]

PUFA/SFA	0.21 b	0.20 b	0.27 a	[25]
	0.12 b		0.23 a	[28]
	0.20			[29]

n-6/n-3	1.72 c	3.77 b	10.38 a	[25]
	2.47 b		5.50 a	[28]
	1.47			[29]
	1.77 b		8.99 a	[30]
	2.78 b		13.60 a	[31]
	1.96 b		3.57 a	[32]

CLA (% total FA)	0.72 a	0.58 b	0.31 c	[25]
	0.33			[33]

IMF: intramuscular fat; SFA: saturated fatty acid; MUFA: monounsaturated fatty acid; PUFA: polyunsaturated fatty acid; CLA: conjugated linoleic acid.

a, b, c mean values in row with different letters differ statistically (*P* < 0.05).
